# Exopolysaccharide Gellan Gum and Derived Oligo-Gellan Enhance Growth and Antimicrobial Activity in *Eucomis* Plants

**DOI:** 10.3390/polym10030242

**Published:** 2018-02-27

**Authors:** Piotr Salachna, Małgorzata Mizielińska, Marcin Soból

**Affiliations:** 1 Department of Horticulture, West Pomeranian University of Technology, 3 Papieża Pawła VI Str., 71-434 Szczecin, Poland; 2 Center of Bioimmobilisation and Innovative Packaging Materials, West Pomeranian University of Technology, 35 Janickiego Str., 71-270 Szczecin, Poland; malgorzata.mizielinska@zut.edu.pl (M.M.); marcin.sobol@zut.edu.pl (M.S.)

**Keywords:** biopolymers, oligosaccharides, pineapple lily, medicinal plants, growth stimulation

## Abstract

One of the visible trends in the cultivation of plants, particularly of medicinal ones, is the increasing interest of researchers in polysaccharides and their derivatives that show biostimulatory properties and are also safe to use. In the current study, we evaluated the effects of gellan gum and its depolymerized form oligo-gellan, on growth and antimicrobial activity of two ornamental species *Eucomis bicolor* and *Eucomis comosa* used in natural medicine. The biopolymers were applied in the form of bulb coating prepared by using polyelectrolyte complexes. In both species investigated, gellan gum and oligo-gellan enhanced the fresh weight of leaves and bulbs, the performance of the photosynthetic apparatus, and the leaf content of basic macronutrients. In comparison with the control, the plants treated with oligo-gellan accumulated more biomass, were first to flower, and had the highest leaf content of potassium. The extracts from the bulbs treated with gellan gum and oligo-gellan showed higher effectiveness in reducing the count of *Bacillus atrophaeus*, *Escherichia coli*, and *Staphylococcus aureus* than those from the bulbs not treated with the polysaccharides. The research described here largely expands our current knowledge on the effects of gellan gum derivatives and has a huge practical potential in agriculture production.

## 1. Introduction

High demand for natural biostimulators improving plant growth and yield encourages researchers to look for new, alternative sources of substances showing substantial biological activity [1]. Natural polysaccharides are a group of compounds with considerable potential in agriculture and horticulture [2,3] used to enhance plant growth and yield [4,5]. Their unique properties include bioavailability, biocompatibility, biodegradability, lack of toxicity, and the ability to form chelates and gels. Thanks to the presence of functional groups, polysaccharides may be subjected to various modifications resulting in the desired biological activity [6]. Particularly important from the practical perspective are depolymerized derivatives of polysaccharides that stimulate plant growth, seed germination, shoot elongation, and root growth, accelerate flowering, enhance gas exchange, antimicrobial activity, and stress resistance, as well as modifying the content of primary and secondary metabolites [7,8,9,10,11]. Research studies showed higher biological effectiveness of chito-oligomers [12], alginate oligosaccharides [13] and oligo-carrageenans [14] in plants as compared with their starting polysaccharides.

Gellan gum is a straight-chain heteropolysaccharide produced by *Sphingomonas paucimobilis* during aerobic fermentation [15]. It contains a repeating unit of a tetrasaccharide consisting of the following monosaccharides: glucose, glucuronic acid, glucose, and rhamnose, so there is a single carboxyl group in each repeating unit [16]. The natural gellan molecule has numerous acyl substituents in its structure which makes it capable of forming soft and elastic gels in the presence of mono- and divalent cations [17]. Gellan gum is used in the food industry [18], pharmaceutical industry [19], for bioremediation of contaminated soils [20], and as a gelling agent in the micropropagation of plants [21]. Previous studies on the effects of gellan gum on plant growth were conducted almost exclusively in vitro. Replacing agar with gellan gum was reported to produce a stimulatory effect on bud regeneration from pear leaves [22]. Similarly, gellan gum-solidified medium improved rooting of microcuttings of difficult-to-root Japanese pear cultivars [23]. In plant tissue cultures of *Eucomis autumnalis* subspecies *autumnalis*, more shoots were obtained on gellan gum than on agar-solidified media [24]. In our earlier work [25] we demonstrated for the first time that the use of gellan gum and chitooligosaccharide in vivo stimulated plant growth and enhanced the content of photoassimilation pigments and mineral nutrients in *Ornithogalum saundersiae*. In the referenced study, polysaccharides were applied in the form of bulb coating before planting, and a patented method for the formation of polyelectrolyte complexes was used [26]. Hydrogel coatings are formed by interaction of anionic functional groups of the polyelectrolyte with metal cations, or by reaction at the interface of aqueous solutions of polyelectrolytes with functional groups of opposite charges [27]. This coating technology based on natural polysaccharides allows for the protection of specific biological materials against negative consequences of their direct exposure to the external environment. An important challenge is to determine the optimal composition of the coatings and their effects on plant growth [28].

Their health-promoting properties and a role in prevention of lifestyle diseases have increased the interest in medicinal raw materials of plant origin [29]. Species belonging to the genus *Eucomis* L’Hér (Asparagaceae family) include the most important medicinal plants used in traditional South African medicine [30]. These plants are used to treat lumbago, rheumatism, stomach pain, and fever, they also support treatment of various respiratory and urinary tract diseases and sexually transmitted diseases [31]. Research publications mostly concern *Eucomis autumnalis*, and antioxidant, antibacterial and antifungal properties of extracts obtained from its bulbs, leaves, and roots [32]. Other *Eucomis* species are also worth investigating, as still little is known of their biological effects [33,34]. Medicinal properties of *Eucomis* resulted in its mass harvesting and its natural populations are now threatened with extinction [35]. Therefore, efficient reproduction methods of *Eucomis* species are being searched for [36,37]. Many species of this genus are also very attractive ornamental plants with a wide range of applications [38,39] but no sustainable methods of their cultivation have been developed so far.

A change in physical and chemical properties, including molecular mass, is a possibility with polymer modification resulting in materials exhibiting new, desired biological activities. To this end, this study investigated the possibility of using exopolysaccharide gellan gum as an effective stimulator of plant growth and the effects of depolymerized gellan gum on growth and antimicrobial activity of *Eucomis bicolor* and *Eucomis comosa*.

## 2. Materials and Methods

### 2.1. Preparation of Oligo-Gellan

Low molecular mass gellan (oligo-gellan) was prepared by acid hydrolysis. A solution of gellan gum (Sigma-Aldrich, Poznań, Poland) was prepared by dissolving 20 g of the substance in 800 mL of deionized water at 70 °C. Then, 36% HCl (Chempur, Piekary Śląskie, Poland) was added to stirred gellan solution to reach a final concentration of 0.2 M. The solution was incubated at 70 °C for 16 h with stirring. After hydrolysis the sample was cooled to room temperature, neutralized with NaOH to achieve pH 7.0 and evaporated to dryness (RVO 200A, INGOS, Praha, Czech Republic). Oligo-gellan was resuspended three times in methanol and filtered. Then the sample was dried at 40 °C for several hours.

### 2.2. High Performance Size Exclusion Chromatography (HPSEC) Analysis

Molecular mass of starting and depolymerized gellan was determined by high performance size exclusion chromatography (HPSEC) using an S1000 pump, an S2300 refractive index detector and a 20 µL sample loop (Knauer, Berlin, Germany). The separation was carried out using SUPREMA 10,000 Å 10 µm column (PSS, Mainz, Germany) at 60 °C and flow rate of 1 mL min^−1^. The eluent composition was 10 mM of disodium edetate in water to avoid gellan aggregation [40] with addition of 0.02% NaN_3_ as antimicrobial agent [41]. Pullulan standards with peak molecular mass 342; 1080; 6100; 9600; 21,100; 47,100; 107,000; 194,000; 344,000; and 708,000 g mol^−1^ (PSS, Mainz, Germany) were used for calibration and relative molecular mass determination. Pullulan standards were prepared at 0.2% concentration, while gellan and oligo-gellan were prepared at 0.5% concentration.

### 2.3. Fourier Transform Infra Red (FT-IR) Analysis

Attenuated total reflectance Fourier transform infrared (ATR-FTIR) spectra were recorded using Spectrum 100 spectrometer, equipped with a diamond ATR crystal (Perkin Elmer Spectrophotometer, Spectrum 100, Waltham, MA, USA), with a resolution of 4 cm^−1^ in the 4000–650 cm^−1^ wavelength range.

### 2.4. Plant Material and Experimental Design

The research was conducted over two growing seasons (2014 and 2015). Each year the bulbs of *Eucomis bicolor* L’Hér. and *Eucomis comosa* Hort. ex Wehrh., with a perimeter of 14–16 cm were purchased from The Netherlands (imported by Ogrodnictwo Wiśniewski Jacek Junior, Warsaw, Poland). Prior to planting, the bulbs were divided into three groups: (I) non-treated bulbs (control), (II) bulbs coated with gellan gum, (III) bulbs coated with oligo-gellan. The coating method was based on polyelectrolyte complexes [26]. The bulbs were soaked for five minutes in 0.5 mass% NaCl used as small molecule ionic gelling compound. After drying (24 h), they were placed for 30 s in 1 mass% solutions of gellan gum or oligo-gellan. The concentration of solution was selected on the basis of our preliminary studies. Then, bulbs were rinsed several times with water and dried for 24 h. Each year a total of 80 bulbs was coated per variant, 20 per each repetition.

### 2.5. Plant Culture

The coated bulbs were planted into individual black PVC pots (18 cm in diameter), filled with deacidified peat (Kronen, Cerkwica, Poland), pH 6.5, supplemented with 3 g dm^−3^ of a multicomponent fertilizer Hydrocomplex (Yara International ASA, Oslo, Norway) containing (mass%) 5 N-NO_3,_ 7 N-NH_4,_ 11 P_2_O_5_, 18 K_2_O, 2.7 MgO, 8 S, 0.015 B, 0.2 Fe, 0.02 Mn, and 0.02 Zn. The pots were placed at random on 60 cm high tables. The plants were grown from mid-March to mid-September under natural photoperiod, in a non-heated tunnel covered with two layers of plastic. They were watered with tap water of the following composition (mg dm^−3^): 1.54 N-NO_3_, 1.4 P, 6.2 K, 98 Ca, 17 Mg, 25 Na, 23 Cl, 0.5 Cu, 0.4 Zn, 1.3 Fe, 195 HCO_3_, electrolytic conductivity 0.64 mS cm^−1^. Mean air temperature during the experiment (2014/2015) was: March 12.1/11.0 °C, April 15.1/13.6 °C, May 18.9/15.4 °C, June 19.7/18.1 °C, July 24.1/20.8 °C, August 19.6/24.1 °C, and September 18.2/17.4 °C

### 2.6. Morphological Trait Assessment

At the beginning of flowering, when the first flowers in inflorescences opened, 10 random plants were selected from each repetition (*n* = 20) and the following traits were determined: total plant height from the soil level to the top of the inflorescence, total number of leaves produced by a single bulb, length of the central leaf in a rosette, number of inflorescences, fresh weight of leaves and bulbs per single plant. Additionally, the number of days from planting the bulbs to the beginning of anthesis was recorded.

### 2.7. Determination of Photosynthetic Parameters

The parameters measured at the beginning of flowering included: leaf greenness index SPAD correlated with chlorophyll content using a Chlorophyll Meter SPAD-502 (Konica Minolta, Osaca, Japan), stomatal conductance with an SC1 porometer (Dekagon Devices, Pullman, WA, USA), net intensity of photosynthesis and leaf transpiration using a LI-COR gas analyzer (Portable Photosynthesis System, Lincoln, NE, USA). The measurements included five plants per treatment. Three well developed leaves from the central section of each plant were selected, four readings per leaf were performed and average values were calculated. The measurements were conducted between 10.00 a.m. and noon, on a cloudless day, at natural CO_2_ level and average PAR 1101 μmol m^−2^ s^−1^ read with a Radiometer-Photometer RF-100 (Snopan, Białystok, Poland).

### 2.8. Nutrient Analysis

To assess plant nutritional status, the content of micro- and macronutrients in the leaves collected from plants at full bloom was analyzed. Five plants per repetition were selected and from each, two fully developed leaves were harvested. The leaves were washed with water, dried up (80 °C for 48 h), and then mineralized. In order to determine the total content of nitrogen, potassium, phosphorus, magnesium, and calcium, the plant material was mineralized for 1 h in 96% H_2_SO_4_ (17 mL for 2 g dry weight). Samples for boron, magnesium, cooper, zinc, manganese, and iron assessment were mineralized for 8 h in a mixture (1:4) of HNO_3_ and HClO_4_ (30 mL for 2 g dry weight). The content of total nitrogen was determined by titration (Kjeldahl method), potassium and calcium by flame photometry, phosphorus and boron by spectrophotometry using a Spectronic GENESYS 6 UV-Visible Spectrophotometer (Thermo Electron Corporation, Cambridge, UK), and magnesium, copper, zinc, manganese, and iron by atomic absorption spectrophotometry (ASS) [42]. The analyses were performed in four repetitions.

### 2.9. Antimicrobial Assay

#### 2.9.1. Bacterial Strains

Three bacterial strains were used in the experiments: *Staphylococcus aureus* (*S. aureus*) strain DSMZ 346, *Bacillus atrophaeus* (*B. atrophaeus*) DSM 675 IZT, and *Escherichia coli* (*E. coli*) DSMZ 498. The strains were obtained from the Leibniz Institute DSMZ-German Collection of Microorganisms and Cell Cultures (Braunschweig, Germany). Acetone (Sigma-Aldrich, Poznań, Poland) was used to extract the active substances from *Eucomis* bulbs harvested at the flowering stage. Antimicrobial properties of the obtained extracts were verified on Tryptic Soy Broth (TSB) and Tryptic Soy Agar (TSA) media (Merck, Darmstadt, Germany). All media were prepared according to a protocol provided by Merck (30 g/L (TSB) or 40 g/L (TSA) was dissolved in purified water and autoclaved 15 min at 121 °C).

#### 2.9.2. Extraction

Dried bulbs from each experimental variant were separately ground to powder and samples of identical mass (5 g) were extracted with 50 g of 70% aqueous acetone. Then, the samples were kept in a sonication water bath for one hour. The temperature of the bath was maintained at 15 °C by adding ice. The acetone extracts were concentrated at 40 °C. After evaporation of acetone the samples were filtered through a 0.2 µm filter. The extracts (15 g of each sample) were used in further analyses.

#### 2.9.3. Antibacterial Activity

Antimicrobial properties of *Eucomis* extracts were checked on TSB and TSA media. First, the cells of *S. aureus*, *B. atrophaeus* and *E. coli* were pre-grown on TSA medium for 24 h at 30 °C. Then, the biomass was suspended in a sterile 0.85% solution of NaCl to achieve 1.5 × 10^8^ colony-forming units (CFU)/mL. After that TSB medium was prepared as well as the double concentrated TSB. 

The next step was to prepare 50% solutions of acetone extracts in 10 mL of TSB; 10 mL of each extract (separately) was introduced into 10 mL of double concentrated TSB.

The suspended biomass was added to sterile flasks with TSB containing extracts at a ratio of 1:10 and stirred with a magnetic stirrer (500 rpm, DragonLab, Beijing, China) for 15 min. The medium with extract-free biomass served as a control. Then the samples were introduced into an incubator/shaker (Ika^®^ KS 4000 i control, Warsaw, Poland) and incubated at 30 °C for 24 h. After incubation/shaking, 100 µL of each sample were plated onto the TSA. Then the decimal dilutions of each sample were prepared and plated onto the TSA. The mediums were incubated at 30 °C for 24 h. Cell concentration was expressed as colony-forming units (CFU) per mL. The results were presented as an average of three samples with standard deviation.

### 2.10. Statistical Analysis

The experiment was a univariate one in a complete randomization arrangement. The results of biometric and physiological measurements and mineral content from two years of the study were subjected to analysis of variance (ANOVA) for univariate experiments. The resulting means were grouped using Tukey’s test. Statistica 13.0 (Statsoft, Cracov, Poland) was used for calculations. The results of antimicrobial assay were presented as an average of three samples with standard deviation.

## 3. Results

### 3.1. Molecular Mass and Fourier Transform Infra Red (FTIR) Spectroscopy

**Figure 1:**
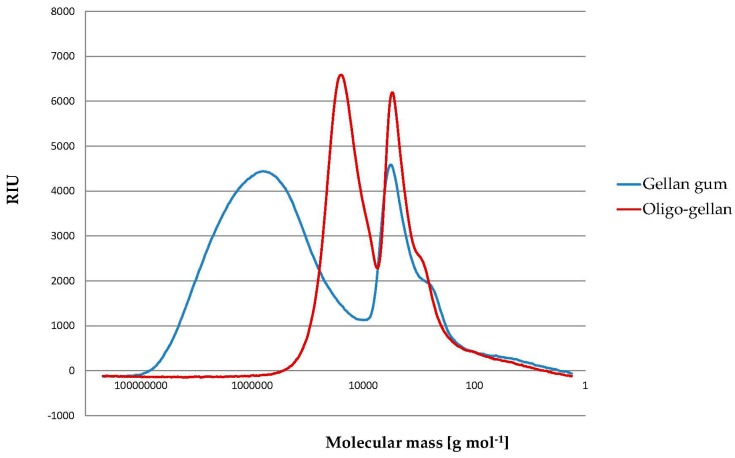
High performance size exclusion chromatography (HPSEC) chromatograms of gellan and oligo-gellan obtained after 16 h of hydrolysis.

Figure 1 shows changes in molecular mass of gellan gum after hydrolysis. Molecular mass of starting gellan gum was 1,000,000 g mol^−1^, and it decreased after 16 h of hydrolysis to 56,000 g mol^−1^.

**Figure 2:**
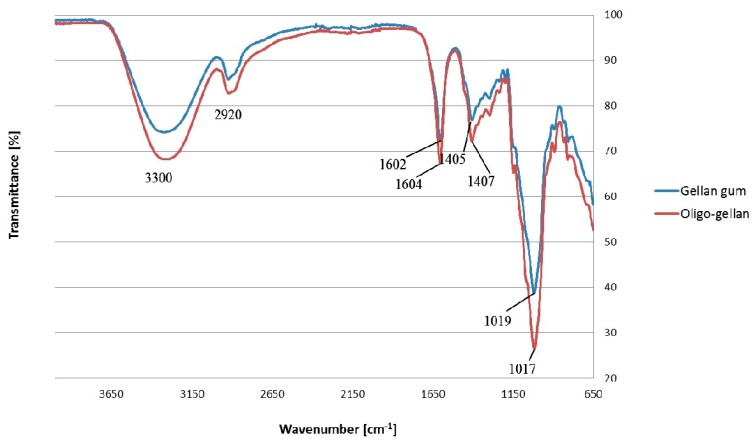
Fourier transform infra red (FTIR) spectra of gellan and oligo-gellan obtained after 16 h of hydrolysis.

Spectroscopic analysis confirmed that the oligo-gellan sample did not undergo chemical modifications during the hydrolysis. Gellan gum and oligo-gellan showed bands (Figure 2) at ~3300 cm^−1^ and ~2920 cm^−1^ that were due to –OH group stretching and C–H stretching, respectively. The peaks at 1602 cm^−1^ for gellan and 1604 cm^−1^ for oligo-gellan were due to an asymmetric COO^−^ stretch, while the bands at 1405 cm^−1^ and 1407 cm^−1^ reflected a symmetric COO^−^ stretch. The peaks at 1019 cm^−1^ and 1017 cm^−1^ were assigned to the C–O stretching for alkyl ether [43].

### 3.2. Growth Attributes

**Figure 3:**
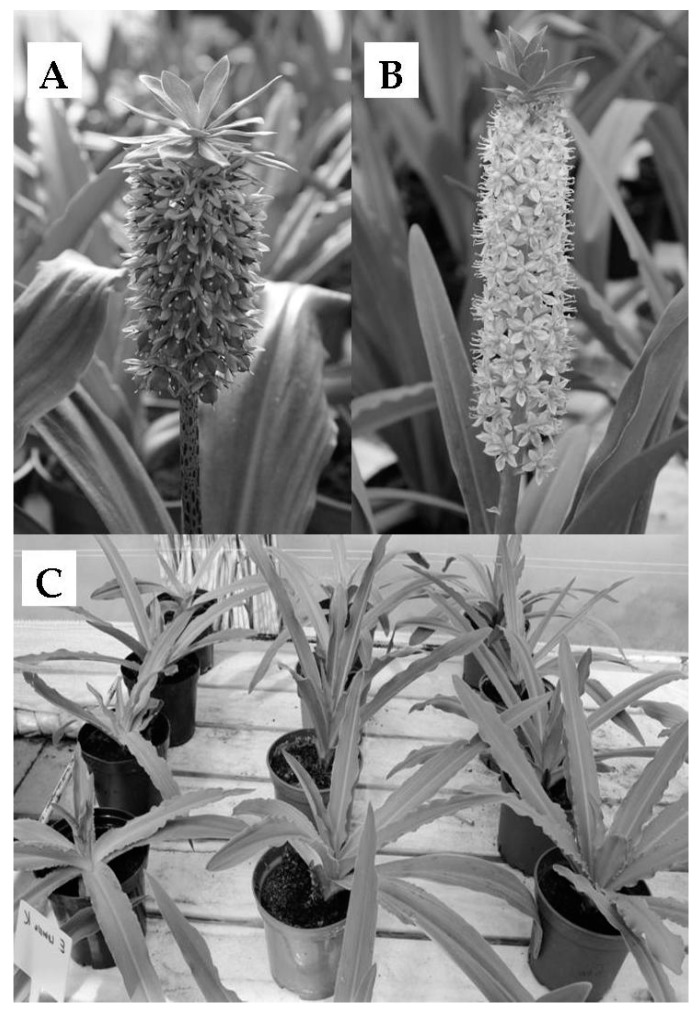
The appearance of *Eucomis bicolor* (**A**) and *Eucomis comosa* (**B**). Visible effects of gellan gum and oligo-gellan on growth of plants (**C**). Left to right: untreated, gellan gum, and oligo-gellan-treated plants.

Bulb coating with gellan gum and oligo-gellan significantly stimulated growth in both *Eucomis* species (Figure 3, Table 1). 

In *Eucomis bicolor* the treatment with gellan gum and oligo-gellan resulted in a higher plant (increased by 9.19% and 11.3%, respectively), longer leaves (by 13.7% and 20.3%), a greater number of leaves (by 5.32% and 4.84%), the fresh weight of leaves (by 11.7% and 25.0%), and the fresh weight of bulbs (by 26.3% and 39.8%), as compared with non-treated control plants. In the second investigated species *Eucomis comosa*, the use of gellan gum and oligo-gellan also increased plant height (by 5.71% and 7.61%, respectively) leaf length (by 10.7% and 12.0%), number of leaves (by 19.8% and 38.0%), fresh weight of leaves (by 12.5% and 27.1%) and the fresh weight of bulbs (by 16.1% and 22.0%). Plants grown from the bulbs coated with oligo-gellan featured considerably greater fresh weight of leaves and bulbs in comparison with those treated with non-depolymerized gellan gum. Additionally, both *Eucomis bicolor* and *Eucomis comosa* treated with oligo-gellan started flowering ten and thirteen days earlier, respectively, than the control plants. The number of inflorescences per plant seemed unaffected by treatment of any of the biopolymers (data not shown).

### 3.3. Physiological Attributes

**Figure 4:**
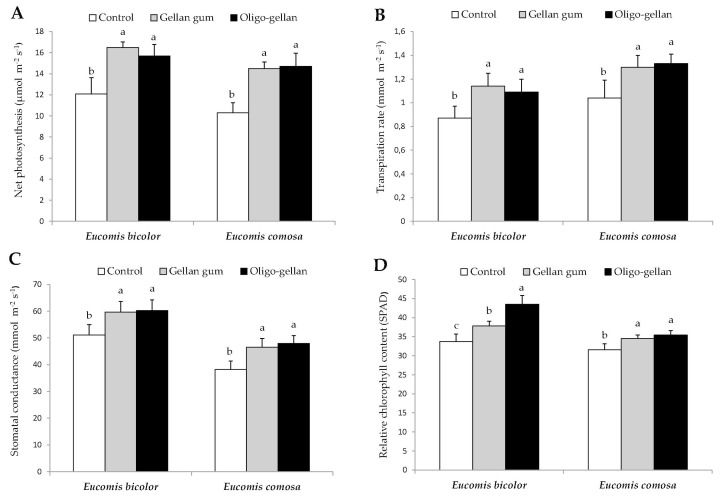
Effect of gellan gum and oligo-gellan on net intensity photosynthesis (**A**), transpiration rate (**B**) stomatal conductance (**C**), and relative chlorophyll content (**D**) in leaves of the two *Eucomis* species. Bars within a chart with the same lower case letter are not significantly different (*p* ≤ 0.05). Values represent the means of four replications ± SD.

Coating the bulbs with gellan gum and oligo-gellan caused a significant improvement in the parameters of photosynthetic apparatus in the leaves of both *Eucomis* species (Figure 4). 

As compared with controls, *Eucomis bicolor* plants treated with gellan gum and oligo-gellan showed increased net intensity of photosynthesis (by 36.4% and 29.8%, respectively), transpiration rate (by 31.0% and 25.3%), stomatal conductance (by 16.8% and 17.8%), and relative content of chlorophyll (by 12.2% and 29.1%). In *Eucomis comosa* coating with gellan gum and oligo-gellan resulted in enhanced net intensity of photosynthesis (by 40.8% and 42.7%, respectively), transpiration rate (by 25.0% and 27.9%), stomatal conductance (by 21.7% and 25.7%) and leaf greenness index (by 9.18% and 12.0%) in relation to non-treated control plants. In general, no significant differences in physiological parameters between the plants grown from bulbs coated with gellan gum and oligo-gellan were observed, except for the relative chlorophyll content which in *Eucomis bicolor* leaves was significantly higher in the variant treated with oligo-gellan.

### 3.4. Leaf Nutrient Concentrations

The study demonstrated that coating the bulbs with gellan gum and oligo-gellan considerably increased the content of some micro- and macronutrients in *Eucomis* leaves (Table 2 and Table 3). The leaves of both species treated with these polysaccharides contained more nitrogen, phosphorus, potassium, boron, and manganese than those in non-treated variants. Moreover, the use of gellan gum and oligo-gellan enhanced leaf content of calcium and zinc in *Eucomis comosa.* A comparison of the effects of gellan gum and oligo-gellan on the content of minerals revealed significantly higher levels of phosphorus, potassium, and calcium in the leaves of *Eucomis comosa* treated with oligo-gellan. Similarly, the leaves of *Eucomis bicolor* grown from the bulbs coated with oligo-gellan contained more potassium and manganese than the leaves of plants grown from the bulbs coated with gellan gum.

### 3.5. Antibacterial Activity

*Eucomis comosa* and *Eucomis bicolor* bulb extracts showed higher activity against Gram (+) than G (−) bacteria (Table 4). The study indicated that *Eucomis bicolor* acetone extracts affected the viability of *S. aureus*. In the medium containing the extract from *Eucomis bicolor* bulbs an average 1 log decrease in the number of bacterial cells was observed. In the case of bulbs non-treated with gellan gum and its derivative, the count of *S. aureus* decreased. The application of gellan gum or oligo-gellan enhanced antibacterial activity of the extract. These data indicated a stimulatory effect of gellan gum and oligo-gellan on antimicrobial properties of *Eucomis comosa* bulb extracts. As emphasized in Table 4, the extracts obtained from the non-treated bulbs did not decrease the count of *S. aureus*. It was also showed that the extracts from *Eucomis comosa* bulbs treated with oligo-gellan exhibited higher activity than those from the bulbs treated with gellan gum.

The study showed that all *Eucomis* extracts affected the viability of *B. atrophaeus*. As illustrated in Table 4, 1 log decrease in the bacterial count was observed for the extracts from *Eucomis bicolor* bulbs that were not treated with gellan gum or oligo-gellan. A 2 log decrease of viable bacteria was noticed for *Eucomis comosa.* The application of gellan gum and oligo-gellan increased the extract activity against *B. atrophaeus*—even 4 log reduction of bacterial count was observed.

The extracts from *Eucomis* bulbs treated with gellan gum and oligo-gellan showed low activity against *E. coli*. Only 1 log decrease of *E. coli* count was noticed. As shown in Table 4, the extracts from bulbs not treated with polysaccharides did not affect the viability of Gram (−) bacteria.

## 4. Discussion

Recent years have witnessed intensive development of research concerning plant production aimed at creating and testing polysaccharides derivatives capable of stimulating plant growth but exerting no negative impact on the environment [44,45]. This paper discusses the results of using a biodegradable polysaccharide gellan gum and its derivative oligo-gellan to coat the bulbs of two *Eucomis* species. It is worth mentioning that this is the first report on the effects of degraded gellan gum on plant growth and development. Our experiments demonstrated a considerable improvement in plant growth, as assessed by morphological and physiological parameters and nutrient content, in plants treated both with gellan gum and oligo-gellan. Bulb coating with the investigated biopolymers resulted in a higher plant, longer leaves, enhanced fresh weight of leaves and bulbs, and accelerated flowering (Table 1). Additionally, the plants of both *Eucomis* species treated with gellan gum and oligo-gellan showed higher performance of the photosynthetic apparatus (Figure 4) and higher levels of nitrogen, phosphorus, potassium, and boron in the leaves (Table 2 and Table 3). These outcomes corroborated our earlier findings in *Ornithogalum saundersiae* where bulb coating with chitooligosaccharide and gellan gum positively affected plant growth and flowering and the content of assimilation pigments and selected nutrients [25]. Gellan gum used as a gelling agent in in vitro cultures improved the yield and morphological behavior of cotyledonary somatic embryos in *Pseudotsuga menziesii* [46], enhanced somatic embryo maturation in *Larix* × *leptolepis* [47] and increased embryogenic potential in *Hevea brasiliensis* [48]. The stimulatory effect of gellan gum and oligo-gellan on plant growth may be due to the presence of nitrogen, protein, uronic acid, minerals, and enzymatic activities exhibited by the biopolymers [22,49]. Also, the reported improved performance of the photosynthetic apparatus in plants treated with gellan gum and oligo-gellan could be associated with increased levels of chlorophyll (Figure 4). Higher content of assimilation pigments is known to intensify photosynthesis, which consequently improves biomass growth and accumulation of minerals. Another reason for the stimulatory effect of gellan gum and oligo-gellan on plant growth might be changes in substrate structure that could be induced by the polysaccharides present in the coating. For example, the alginic acid salts bind with metal ions in the soil and form a complex that absorbs moisture and improves the substrate structure in the root zone [50]. This improves aeration and results in a favorable number of capillary pores, which in turn stimulate the root system growth and positively affect soil microflora. As a result, plants may more efficiently uptake water and other nutrients [51,52]. Further studies seem necessary to better understand the mechanism of action of the investigated polysaccharides.

Our study compared the biological activity of gellan gum and oligo-gellan. Both biopolymers were found to stimulate growth of the investigated *Eucomis* species but the plants treated with oligo-gellan produced leaves and bulbs of greater fresh weight, flowered earlier, and accumulated higher levels of potassium. These outcomes confirmed previous findings that indicated positive effects of oligomers obtained from degraded polysaccharides on the growth and yield of different plant species such as *Capsicum frutescens* [53], *Trigonella foenum-graecum* [54], and *Eucalyptus globulus* [55]. The improved examined growth parameters as a result of gellan gum and oligo-gellan application might be ascribed to the role of oligosaccharides in plant growth stimulation in general [56]. It is well known that oligosaccharides are important signaling molecules that mediate a variety of cellular processes, including growth, organogenesis, and survival of plants in unfavorable environments by exploiting gene expression [57].

The extracts from bulbs treated with gellan gum and oligo-gellan showed higher activity than the extracts from bulbs that were not treated with the polysaccharides (Table 4). It is tempting to suggest that gellan gum and its derivative improved the antimicrobial properties of *Eucomis* extracts. Gellan gum and oligo-gellan enhanced the activity of bulb extracts against *E. coli*. A decrease of G (−) bacteria growth was noticed only for the extracts obtained from plants treated with polysaccharides. It may be concluded that gellan gum and oligo-gellan had a positive effect on the antimicrobial properties of *Eucomis* extracts against both G (+) and G (−) microorganisms. Stronger antimicrobial activity of the extracts from *Eucomis* bulbs treated with gellan gum and its depolymerized form may be due to the positive effects of the polysaccharides on the content of secondary metabolites, as demonstrated in *Ornithogalum saundersiae* [25]. In that study, plants grown from bulbs coated with chitooligosaccharide and gellan gum featured increased content of total polyphenols, l-ascorbic acid, and antioxidant activity. Similarly, an application of degraded polysaccharides enhanced the accumulation of some polyphenolic compounds with potential antimicrobial activity in *Eucalyptus globulus* [55], total phenolics in *Mentha arvensis* [58], and artemisinin in *Artemisia annus* [59], as well as increasing essential oil yield and the content of active constituents in *Foeniculum vulgare* [60]. Our current knowledge does not offer comprehensive answers to the question of how the degraded polysaccharides affect plant metabolism. Therefore, further studies, including the analysis of transcript and protein levels, are recommended to elucidate the mechanisms responsible for stimulatory effects of gellan gum and oligo-gellan on plant growth and content of biologically active substances.

## 5. Conclusions

The study demonstrated stimulatory effects of both gellan gum and oligo-gellan, used for coating of *Eucomis bicolor* and *Eucomis comosa* bulbs, on the morphological traits, biomass, photosynthesis performance, and accumulation of basic macronutrients such as nitrogen, phosphorus, and potassium in plant leaves. The treatment of *Eucomis comosa* and *Eucomis bicolor* bulbs with gellan gum and oligo-gellan enhanced the activity of bulb extracts against G (+) and G (−) bacteria. Oligo-gellan was more effective in increasing the fresh weight of leaves and bulbs, which may be particularly important when the species are grown for herbal extracts. The study enabled also the improvement of the cultivation technology and quality of *Eucomis* plants. However, the mechanisms of action of gellan gum and oligo-gellan on growth and development in plants remain unknown. Further studies will be necessary in order to solve these problems.

## Figures and Tables

**Table 1:** Effect of gellan gum and oligo-gellan on growth parameters of the two *Eucomis* species. Values are presented as means ± SD. Mean values followed by different letters in each column were significantly different (*p* ≤ 0.05).

Treatment	Growth Parameters					
	Plant Height (cm)	Leaf Length (cm)	Number of Leaves	Days to Anthesis	Leaves Fresh Weight (g)	Bulbs Fresh Weight (g)
	***Eucomis bicolor***					
Control	56.6 ± 2.61 ^b^	28.6 ± 1.49 ^b^	6.20 ± 0.17 ^a^	80.8 ± 1.89 ^a^	128 ± 9.17 ^b^	118 ± 6.03 ^c^
Gellan gum	61.8 ± 1.87 ^a^	32.5 ± 1.15 ^a^	6.53 ± 0.06 ^a^	75.0 ± 1.95 ^b^	143 ± 8.74 ^a,b^	149 ± 5.57 ^b^
Oligo-gellan	63.0 ± 1.27 ^a^	34.4 ± 1.10 ^a^	6.50 ± 0.20 ^a^	71.3 ± 1.31 ^c^	160 ± 6.66 ^a^	165 ± 7.09 ^a^
	***Eucomis comosa***					
Control	47.3 ± 1.67 ^b^	46.6 ± 0.85 ^b^	12.1 ± 1.01 ^b^	87.3 ± 1.12 ^a^	144 ± 10.1 ^b^	205 ± 5.57 ^b^
Gellan gum	50.0 ± 1.30 ^a^	51.6 ± 1.83 ^a^	14.5 ± 0.50 ^a^	81.3 ± 0.91 ^b^	162 ± 6.08 ^a,b^	238 ± 17.3 ^a,b^
Oligo-gellan	50.9 ± 1.07 ^a^	52.2 ± 2.36 ^a^	16.7 ± 1.12 ^a^	74.4 ± 1.30 ^c^	183 ± 10.4 ^a^	250 ± 15.0 ^a^

**Table 2:** Effect of gellan gum and oligo-gellan on macronutrient concentrations (% dry weight) in leaves of the two *Eucomis* species. Values are presented as means ± SD. Mean values followed by different letters in each column were significantly different (*p* ≤ 0.05).

Treatment	Macronutrients				
	Nitrogen	Phosphorus	Potassium	Magnesium	Calcium
	***Eucomis bicolor***				
Control	2.03 ± 0.15 ^b^	0.24 ± 0.08 ^b^	1.66 ± 0.12 ^c^	0.18 ± 0.01 ^a^	2.56 ± 0.05 ^a^
Gellan gum	2.70 ± 0.10 ^a^	0.62 ± 0.16 ^a^	2.30 ± 0.19 ^b^	0.18 ± 0.01 ^a^	2.57 ± 0.09 ^a^
Oligo-gellan	2.66 ± 0.05 ^a^	0.70 ± 0.15 ^a^	2.63 ± 0.21 ^a^	0.18 ± 0.01 ^a^	2.60 ± 0.01 ^a^
	***Eucomis comosa***				
Control	2.23 ± 0.21 ^b^	0.29 ± 0.02 ^c^	3.20 ± 0.20 ^c^	0.16 ± 0.01 ^a^	2.11 ± 0.16 ^c^
Gellan gum	3.24 ± 0.15 ^a^	0.40 ± 0.05 ^b^	3.51 ± 0.19 ^b^	0.17 ± 0.02 ^a^	2.23 ± 0.20 ^b^
Oligo-gellan	3.34 ± 0.14 ^a^	0.57 ± 0.08 ^a^	3.88 ± 0.22 ^a^	0.17 ± 0.02 ^a^	2.62 ± 0.11 ^a^

**Table 3:** Effect of gellan gum and oligo-gellan on micronutrient concentrations (mg kg^−1^ dry weight) in leaves of the two *Eucomis* species. Values are presented as means ± SD. Mean values followed by different letters in each column were significantly different (*p* ≤ 0.05).

Treatment	Micronutrients				
	Boron	Copper	Zinc	Manganese	Iron
	***Eucomis bicolor***				
Control	15.7 ± 1.75 ^b^	2.92 ± 0.33 ^a^	35.7 ± 2.05 ^a^	46.3 ± 2.71 ^b^	64.9 ± 6.31 ^a^
Gellan gum	36.7 ± 3.30 ^a^	2.81 ± 0.29 ^a^	35.8 ± 4.11 ^a^	51.8 ± 2.71 ^a^	65.3 ± 9.97 ^a^
Oligo-gellan	33.0 ± 3.18 ^a^	2.82 ± 0.28 ^a^	35.6 ± 2.35 ^a^	52.7 ± 3.12 ^a^	66.0 ± 7.23 ^a^
	***Eucomis comosa***				
Control	23.8 ± 4.10 ^b^	2.21 ± 0.11 ^b^	34.8 ± 2.82 ^b^	32.1 ± 1.85 ^b^	75.2 ± 5.46 ^b^
Gellan gum	35.2 ± 2.33 ^a^	3.11 ± 0.41 ^a^	39.4 ± 0.71 ^a^	34.1 ± 2.32 ^a,b^	84.9 ± 15.6 ^a,b^
Oligo-gellan	34.7 ± 3.29 ^a^	3.18 ± 0.33 ^a^	39.0 ± 1.00 ^a^	40.1 ± 4.56 ^a^	100 ± 12.0 ^a^

**Table 4:** The effect of gellan gum and oligo-gellan on the viability of Gram (+) and Gram (−) microorganisms of 50% acetone extracts of *Eucomis* bulbs.

Treatment	Concentration of Bacterial Cells		
	*S. aureus* (10^−7^ CFU/mL)	*B. atrophaeus* (10^−7^ CFU/mL)	*E. coli* (10^−6^ CFU/mL)
	***Eucomis bicolor***		
Control	63.67 ± 10.69	12.70 ± 0.57	32.00 ± 5.00
Gellan gum	29.20 ± 1.39	9.17 ± 0.64	2.43 ± 0.32
Oligo-gellan	16.58 ± 1.22	7.93 ± 0.64	3.17 ± 0.45
	***Eucomis comosa***		
Control	148.17 ± 13.08	1.26 ± 0.06	34.00 ± 5.13
Gellan gum	41.17 ± 1.10	0.95 ± 0.05	6.13 ± 0.25
Oligo-gellan	8.47 ± 0.66	0.09 ± 0.02	5.57 ± 0.40

Trypticasein soy broth (TSB) bulion devoid of extract: *S. aureus* = 199.0 ± 91.65 *×* 10^−7^ CFU/mL; *B. atrophaeus* = 199.00 ± 9.17 *×* 10^−7^ colony-forming units (CFU) per mL; *E. coli* = 46.3 ± 2.52 *×* 10^−6^ CFU/mL.
